# 

**Published:** 1889-02-02

**Authors:** 


					PERMANENTLY ENLARGED TO 32 PAGES.
.PRICE ONE PENNY. . . . . . . /?>
tfo. 123, Vol. v.-
-February 2nd, 1889.
W\I
FLjWf )|g^ Per Annum, 6s. 6d., post free. U
tHegjUtered at tlie General Post O/lice " ~ JL* M1M, JEL
act a Newspaper.] '?
Monthly Parts. 6d. ; post free. 7id.
Ditto per Ann. 7s. 6d., post free.
A Weekly Institutional Journal of
Qctnur, /Iftebtchtc, IRtustng, airir fl^ilautljMpg.

				

